# The structure of human olfactory space

**DOI:** 10.1186/1471-2202-14-S1-P323

**Published:** 2013-07-08

**Authors:** Alexei Koulakov, Dmitry Rinberg

**Affiliations:** 1 Cold Spring Harbor Laboratory, Cold Spring Harbor, NY, 11724, USA; 2 New York University, New York, NY, 10016, USA

## 

**Figure 1:**
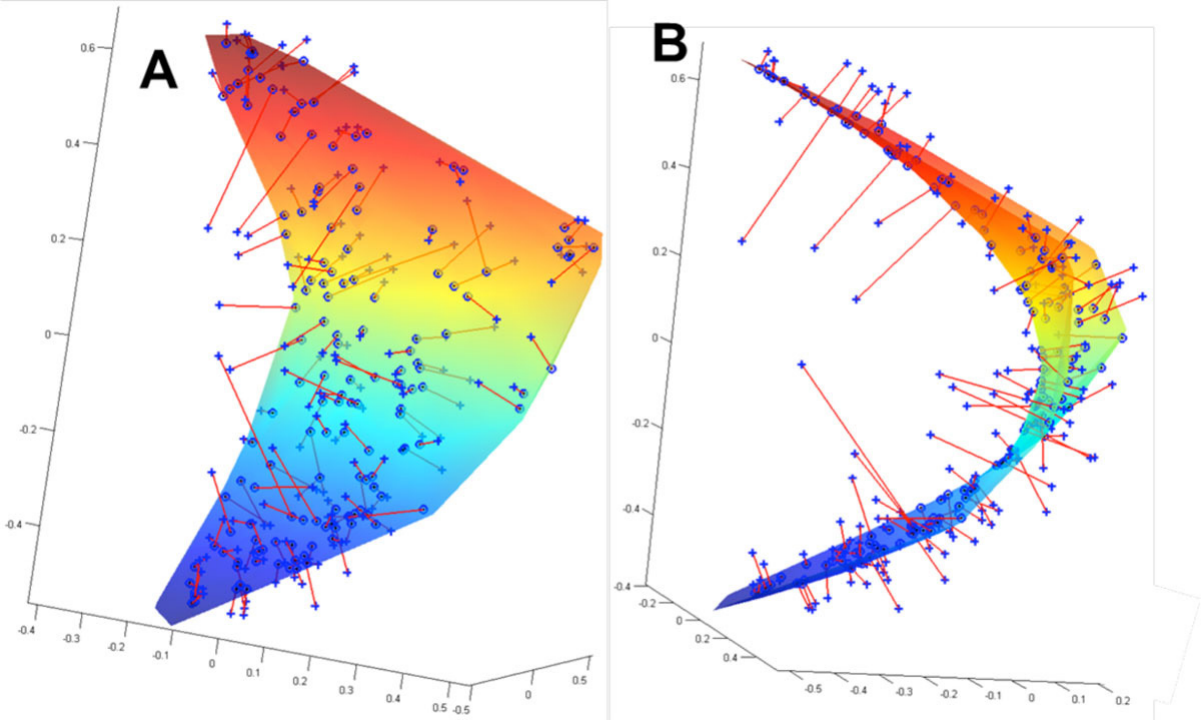
**Odorants in the perceptual space**. (A) Each of the 144 odorants can be represented as a point in the 146D space of perceptual (semantic) descriptors. The odorants are shown by blue crosses placed in this projection onto the 3D space of principal components. (B) When viewed from a certain direction, the odorants clustered near a C-shaped 1D curve, suggesting that in 3D the odorants are distributed close to a 2D curved surface. The 2D surface shown represents the best fit to the data. The odorants (blue crosses) are connected to the nearest points on the surface by the red lines representing the residual errors. The 2D surface minimizes the total squared length of the residuals computed in 146D. The total squared length of residuals can be viewed as the remaining variance in the data not accounted for by the projection onto the 2D curved manifold. The residual variance constitutes <50% of the total variance in the case of 2D curved manifold.

We analyze the responses of human observers to an ensemble of monomolecular odorants and mixtures. Each odorant is characterized by a set of 146 perceptual descriptors obtained from a database of odor character profiles. Each odorant is therefore represented by a point in a highly multidimensional sensory space. In this work, we study the arrangement of odorants in this perceptual space. We argue that odorants densely sample a two-dimensional curved surface embedded in the multidimensional sensory space (Figure 1). This surface can account for more than half of the variance of the perceptual data. We also show that only 12% of experimental variance cannot be explained by curved surfaces of substantially small dimensionality (<10). We demonstrate that the space of mixtures can be approximated by the monomolecular curved manifolds of similar dimensionality, thus suggesting that the geometry of the perceptual space is similar for both mixtures and monomolecular odorants. We propose that these curved manifolds represent the relevant spaces sampled by the human olfactory system, thereby providing surrogates for olfactory sensory space. For the case of 2D approximation, we relate the two parameters on the curved surface to the physico-chemical parameters of odorant molecules. We show that one of the dimensions is related to eigenvalues of molecules' connectivity matrix, while the other is correlated with measures of molecules' polarity. For a higher dimensional curved manifold, we establish mapping between the space of odorant physico-chemical properties and the coordinates in the olfactory space. This allows building *de novo *predictor for the odorant perceptual quality on the basis of a molecule's structure. This method proves accurate for a substantial fraction of tested monomolecular odorants.

